# Implications of digital fertility tracking for clinical care: a qualitative systematic review

**DOI:** 10.1186/s12978-025-02083-1

**Published:** 2025-10-06

**Authors:** Kathryn Sheridan Clay, Tori Ford, Rosa Mackay, Sabrina Keating, Sue Ziebland, John Powell

**Affiliations:** 1 Nuffield Department of Primary Care Health Sciences, University of Oxford, Radcliffe Primary Care Building, Radcliffe Observatory Quarter, Woodstock Rd, Oxford, OX2 6GG UK; 2 Nuffield Department of Primary Care Health Sciences, University of Oxford, Oxford, UK

**Keywords:** Infertility, mHealth, Fertility monitoring, Health-tracking, Smartphone

## Abstract

Research on the use of digital health interventions for the management of infertility is still emerging and remains understudied. This review syntheses cross-domain qualitative research on the use of digital fertility trackers. We identified 29 papers and thematic analysis found that these tools are most frequently used alongside, but also sometimes in place of clinical care. The research shows that they pose significant disruption to patient-provider relationships and the broader fertility industry and may place patients at risk when developed without a strong research or medical base, or if used incorrectly. More work is needed on the impact of these tools on care pathways, and to provide guidance on differentiating evidence-based platforms from low quality trackers to safeguard patients and improve fertility treatment outcomes.

## Abstract (highlights)

Research on using digital technologies such as apps to manage infertility is still new and not well studied. This literature review looked at 29 studies and articles on how people use digital fertility trackers either alongside or instead of fertility care in clinic or hospital settings. While these tools are often used by people in tandem with care from a healthcare professional, authors suggest they disrupt the relationship between patients and providers and may be changing the fertility industry itself. If these tools are poorly designed or used incorrectly, they can put patients at risk. More research is needed to understand how these tools affect fertility care and to help people and healthcare professionals identify high-quality trackers that are safe and effective.

## Introduction

Fertility management products are some of the most popular mobile applications in terms of downloads within the women’s health and wellbeing category (also known as ‘Femtech’), and are pioneering virtual services through telehealth, at-home lab testing, and device integration with fertility tracking wearables [1]. Literature exploring these tools has emerged in the areas of Medical Sociology, Gender Studies, Law, and Science and Technology Studies. However, few existing studies or systematic reviews focus on the use of digital health tools for trying to conceive in the context of fertility treatment. The aim of this review is to evaluate and summarise cross-domain emerging research on fertility trackers, with particular attention to how they are used in tandem or in place of clinical fertility treatment.

## Background

Existing qualitative research analyses and summarises the legal and regulatory environment of fertility trackers, user experiences and use of tools, and how tools are shaping the ways users experience their fertility journey. The two major existing reviews identify the types of digital tools available for infertility patients [2, 3]. The reviews describe the types of features and costs, what needs the tools are developed to address, and discuss thematic analyses of user reviews. A search of the PROSPERO database found one ongoing review registered in 2020 on the usage and effectiveness of family planning digital health interventionstargeting individuals or health care providers, but there are no further updates, publications, or conference papers linked to the study protocol.

Several reviews have sought to answer more specific questions about the use of self-tracking technologies for fertility but lack a systematic approach. Meyers and Domar completed a broad literature search in 2021 on digital tools designed to reduce the emotional toll of infertility, while Alfawzan and colleagues completed a review of the top 23 mHealth applications and assessed their data sharing policies and practices in 2022 [4, 5]. One 2021 scoping review has been completed on how menstruation and fertility apps are used by patients, with a third of the papers reviewed related to fertility trackers, and noted a limited evidence base within the field [6]. While these works provide a helpful overview of the types, uses, features, and privacy policies of existing fertility trackers, this review seeks to address the gap in syntheses focused on their positioning within or outside clinical health care services.

## Methods

A systematic literature search was conducted to identify research published on the use of digital fertility trackers to assist individuals trying to conceive. The authors published our search protocol to PROSPERO (registration number CRD42024491336).

### Search strategy

**Fig. 1:**
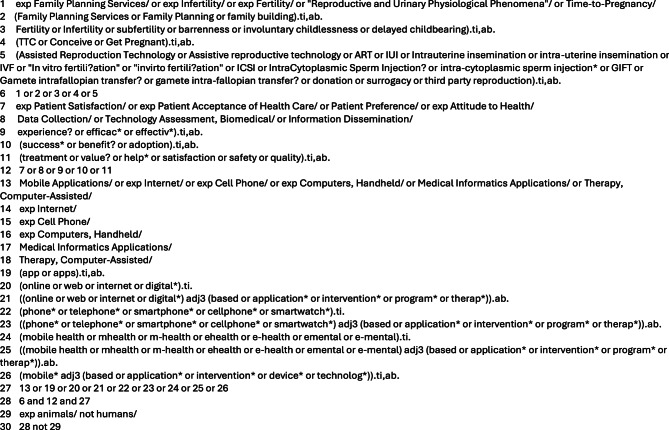
Example ovid search (image by author)

The search strategy and key search terms for this paper were developed with assistance from a librarian specialized in health research. Feedback from two patient and public involvement (PPI) members helped to expand our inclusion of lay words for infertility. A search was conducted of the following databases: Cochrane Library, Ovid MedLine, and EMBASE (see Fig. 1 as example). Since research in this area is limited, we also included gray literature to provide a more comprehensive examination of published material as recommended by Cochrane [7]. Grey literature was sought by using key terms such as “infertility”, “conception”, and “digital health” through Google Scholar, SCOPUS, and resources listed on the Public Health England Index of Grey Literature Sources. Our inclusion criteria required grey literature to be published either as conference proceedings, as a thesis, or in a peer-reviewed journal to limit the search scope to academically rigorous research.

References of selected papers and related literature reviews were also searched for additional citations. The search was conducted in March of 2024.

### Screening, quality assessment, and data analysis

**Fig. 2:**
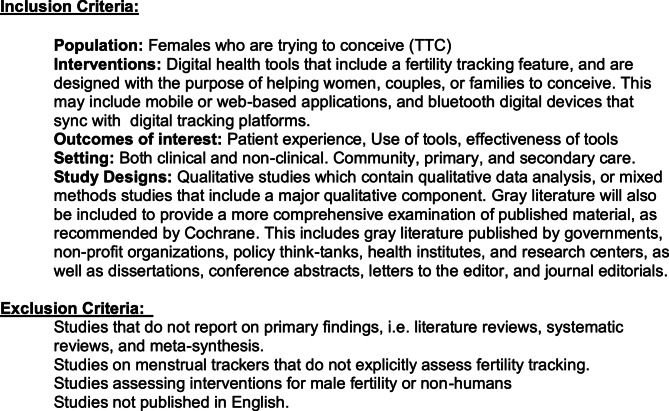
Inclusion and exclusion criteria (image by author)

Studies were included if they were focused on people assigned female at birth trying to conceive (TTC) who were using digital health tools designed with a fertility tracking feature for the purpose of conceiving (see Fig. 2 below for full inclusion and exclusion criteria).

Our review team used the Covidence software to manage citations, remove duplicates, track conflicts between reviewers, note reasons for exclusion, complete a quality review using the Critical Appraisal Skill Programme (CASP) structure, and complete data extraction (see image 1). Titles of papers and abstracts were screened, full texts reviewed, and screening conflicts resolved by KSC, TF, SK, and RM. Quality screening was conducted by KSC with review and discussion with TF and RM. SZ and JP were consulted to resolve conflicts in which the authors could not find consensus. No articles were excluded based on their quality, as we aimed to consolidate all relevant research on this topic to provide a comprehensive mapping of the literature. This allowed us to include a broader range of research to add to the descriptive breadth of the work. For transparency, quality is reported in Table 1: Summary of Included Literature. Once the summary data from the papers was extracted, NVIVo was used to code and compile themes. KSC led initial coding in NVivo, followed by review, discussion, and consensus with TF, RM, SZ, and JP.

**Table 1:** Summary of included literature

Author	Country	Aim/Technology	Methods	Key Findings	Industry Funded?	Quality Summary
Allard-Phillips et al. 2023 [8]	United States	Association between fertility tracking app use and quality of life	Cross-sectional survey *P* = 149	Most patients surveyed presenting for new infertility visits at a fertility clinic reported using a fertility mobile app and found them helpful	X	Poor methodological rigor: qualitative component of the study had undefined and undefended methods, no description of how long survey was live, or which patients were sampled. Low transparency in how qualitative data was analysed. Included as grey literature, but limited information in the conference abstract.
Bench-Capon et al. 2018 [9]	United Kingdom	See how women perceive the fertility predictions that are provided through calendar-based cycle tracking apps	Thematic analysis of interviews with women who owned a smartphone and were trying to conceive *P* = 38	Many women reported calendar-based apps incorrectly estimated their ovulation window	✔	Poor methodological rigor and analysis transparency: little discussion of the potential for bias, no information on whether data saturation was reached, or how participants were recruited. Included as grey literature, but limited information available in the conference poster.
Blair et al. 2021 [10]	United Kingdom	Explore how women trying to conceive use fertility tracking apps, including in combinations with ovulation tests like Clearblue	Thematic analysis of interviews with women trying to conceive using fertility tracking apps *P* = 24	Nearly all respondents were aware of fertility tracking apps and reported they would use them to try and conceive	✔	Strong methodological rigour and transparency; qualitative approach, appropriateness of design, and data saturation is discussed. The recruitment strategy potentially introduces bias and reduces generalisability, since participants were recruited through SPD Clearblue consumers and were not ethnically or geographically diverse.
Costa Figueiredo, et al. 2017 [11]	United States	Understand how women engage in tracking fertility information, and the challenges involved	Qualitative analysis of patient-generated content in an online health community dedicated to fertility issues *P* = 400 posts	Women use self-tracking throughout their fertility journey, but report it can be a complex and emotionally burdensome process	X	Well justified exploratory qualitative design, data collection included threads were randomised and all stages of analysis well documented to enhance transparency. However, collected data from an online health forum, which biases results to women willing and able to share experiences online and may limit validity and reliability of findings.
Costa Figueiredo 2018 [12]	United States	Explore how fertility self-tracking activities characterise how people engage with personal health data	Qualitative analysis of patient-generated content in an online health community dedicated to fertility *P* = 100 posts	People engaging with fertility self-tracking data experience both positive and negative experiences, but many find self-tracking can reinforce negative feelings	X	Clear explanation of the research design and choice of personal informatics model, increasing methodological rigor. Strong transparency in how data was collected and classified, as well as limitations, for reproducibility. However, collected data from an online health forum, which biases results to women willing and able to share experiences online and may limit validity and reliability of findings.
Costa Figueiredo 2020 [13]	United States	Evaluate how users perceive and trust commercial fertility apps	Qualitative evaluation of fertility app algorithms and user reviews *P* = 30	Users report mixed reactions towards ovulation tracking apps, with some fully trusting predictions, but many reporting inaccuracies	X	Though the analysis is described as qualitative, results are mainly present quantitatively which makes interpreting qualitative data difficult. Data saturation is not discussed, and limited information on thematic analysis. Collected data from online user reviews, which may bias results to users who feel strongly enough (either positively or negatively) to provide reviews online. Reviews may also become outdated with app updates. Included as grey literature, but limited information in the conference abstract.
Costa Figueiredo 2021 [14–16]	United States	Study the use of patient generated health data and its practices by patients and clinicians	Thematic analysis of interviews with patients facing challenges to conceive and fertility clinic providers *P* = 19	Patients and providers value different fertility-tracking data, but both appreciate tracking data for its use in fertility care.	X	Strong transparency in the reporting of recruitment methods and data collection, and strong detail in methods which contribute to rigor. However, exclusive recruitment of healthcare providers from a single clinic limits the transferability of the findings, and data saturation is not discussed, raising concerns about the reliability and completeness of discussed themes.
Costa Figueiredo 2021 [14–16]	United States	Analyse (in)fertility experiences with fertility data tracking and technology	Qualitative analysis using Ecological Systems Theory of interviews with patients, partners, and health providers *P* = 26	Patients and providers view fertility data as personal and private, but it also influences and is influenced by close relationships, institutions, and sociocultural contexts.	X	Strong methodological rigor underpinned by a thorough justification of the study design and theoretical framework. High transparency throughout the data analysis, with clear articulation of themes and coding methods, and strong data to support categories with transparency around conflicting data, increasing credibility. However, low diversity across the participant sample reduces the transferability of results.
Costa Figueiredo 2021 [14–16]	United States	Identify how people’s planned, expected, and unexpected life stages, events, and transitions impact how apps support their tracking behaviours	Evaluation of thirty-one top fertility apps app store pages, app features, and user reviews *P* = 15 apps	Popular fertility tracking apps support specific, standardised conception goals without considering holistic and personalised aspects of tracking.	X	Strong methodological rigor in that saturation is well discussed, the sample is large, and data handling and research design are both transparently described. However, collected data from online user reviews, which may bias results to users who feel strongly enough (either positively or negatively) to provide reviews online, reducing validity and reliability of findings. Questionable study design appropriateness given the objectives and aims of the research. Included as grey literature, but limited information in the conference abstract.
Costa Figueiredo 2024 [17]	United States	How app descriptions influence individuals’ attitudes towards algorithmic recommendations in fertility self-tracking	Qualitative analysis of survey and Kaya app simulation *P* = 298	Participants prefer and have more trust in fertility self-tracking app descriptions that include AI descriptions.	X	Strong methodological rigor through transparent discussion of intervention development and reporting of diverse participant demographics, including attrition. However, the authors developed the tools undergoing tested, introducing potential researcher bias and limiting credibility. Participants were only briefly introduced to and used the tested tool, which may impact completeness of data. Reliance on non-academic sources in the findings citations also reduces overall credibility.
Donelle 2021 [18]	Canada	Understand the ways digital technologies contribute to the experience of the transition to parenting	Thematic analysis of focus groups and interviews with new parents’ experiences with and uses of digital technology *P* = 25	Participants reported frequent use of digital technologies to inform their fertility journeys but expressed concerns about lack of information and negative impacts of use.	X	The study demonstrates strong methodological rigor through high transparency in its study design and description of focus groups and interviews. Data analysis transparency was strong with a detailed description of the process, the achievement of data saturation, robust support of themes by rich quotes, and discussion of contrasting data. The researchers also show strong reflexivity throughout the text, increasing credibility of findings. However, the lack of participant diversity and only a sub-section of interviewees trying to conceive impacts the transferability of findings.
Freilich 2022 [8]	United Kingdom	Explore the role of the Natural Cycles App as an emerging contraceptive method and a means of menstrual tracking	Thematic analysis of interviews of Natural Cycle users *P* = 30	Participants most likely to find the app useful kept strict routines and were ambivalent about trying to conceive. Individuals reported the app reinforced gendered norms and expectations.	✔	The lack of participant diversity and recruitment though an existing app (Natural Cycles) limits methodological rigor and validity of findings Most interviews were with participants trying to avoid conception, so only a subset of data (i.e. interviews with those who had switched to trying to conceive) were relevant to this review, limiting the appropriateness of study design for this review’s aim. As an unpublished thesis it has not undergone peer-reviewed which may limit credibility, but included as grey literature.
French 2022 [19]	United Kingdom	Explore how women and their partiers navigate preconception healthcare and the role of Natural Cycles in this process	Thematic analysis of interviews with women and partners of women using Natural Cycles *P* = 30	Women reported conversations about trying to conceive were challenging in healthcare settings, and felt the app improved their understanding of their fertility.	✔	Demonstrates methodological rigor through a clear description of data coding practices and when data saturation was reached. Strong detail on the topic guide used increasing data collection transparency. However, most participants were financially secure and had a high level of education, potentially biasing findings and reducing their transferability.
Gambier-Ross 2018 [20]	United Kingdom	Explore women’s uses of and relationships with fertility tracking apps	Mixed methods evaluation (survey and interviews) of women’s experiences with fertility tracking apps *P* = 11	Women who used fertility tracking apps often used them to try and conceive or to inform fertility treatment. However, many reported necessary design changes to make the tools more useful.	X	Shows methodological rigor by clearly defending the research design and improved data collection transparency by describing recruitment materials. Transparency in the data analysis was robust, with saturation reached, and clear presentation of subthemes and coding framework. Only a minority of survey respondents used a fertility tracking app, and the survey was only online for five days, which may reduce the generalisability of the data. Only a subset of survey information (i.e. related to TTC) was useful to this review due to the study design.
Grenfell 2021 [21]	United Kingdom	Explore the experiences of cisgender women and partners with Natural Cycles in the context of their daily lives	Thematic analysis of interviews with people using the Natural Cycles app to conceive *P* = 30	Women viewed fertility trackers as “natural”, less obstructive means of controlling their reproductive health, though many apps also reinforced gendered responsibilities and anxieties.	✔	The study design is well described, particularly theoretical lenses and objectives, and the use of purposive sampling discussed, defending the appropriateness of the approach. Coding process is clear, topic guide is discussed, and contrasting data included which improve the transparency of the data collection and analysis. The study is solely focused on one app, limiting generalisability and transferability of findings. Additionally, the very brief and short use of quotes may reduce context, introducing researcher bias and negatively impacting credibility of the thematic findings.
Hamper 2020 [22]	United Kingdom	Women’s use of fertility tracking apps while trying to conceive	Qualitative analysis of interviews with women using fertility apps to conceive *P* = 15	Fertility tracking apps actively impact women’s sense of self, identity, and view of their reproductive bodies.	X	Robust descriptions of ontological stance, theoretical application, and recruitment choices showed strong methodological rigor. However, the interview participants were largely white and middle class, limiting transferability and generalisability. The study lacks a description of the topic guide, how interviews were conducted, or whether data saturation was reached, negatively impacting the study’s transparency and credibility.
Hamper 2022 [14]	United Kingdom	Explore heterosexual wo men’s experiences of using fertility apps in the context of trying to conceive	Qualitative analysis with women familiar with or using fertility tracking apps to assist with conception *P* = 16	Though fertility apps with shared tracking features attempt to redistribute the reproductive labour involved with fertility self-tracking, socio-cultural expectations around gender and conception significantly limit their success.	X	Study design is clearly described, critical theory is well detailed, and in-depth interview data reported which contribute to the rigor of the study. Rich data is included to validate themes, and strong reflexivity is used, particularly around the use of laughter during interviews, increasing credibility. However, interview participants were largely highly educated, middle class, and aligned with heteronormative ideals, reducing transferability and generalisability. Additionally, low transparency on how themes and codes were organised, and whether a topic guide was used reduce the credibility of the study.
Harrison 2023 [23]	United Kingdom	Assess possibility of designing a fertility treatment intervention acceptable and feasible to both patients and healthcare professionals	Thematic analysis of online interviews with healthcare providers and patients *P* = 26	Both patients and providers felt a digital intervention to support assisted reproductive planning would be beneficial, though difficult to implement into current clinical practice.	✔	High transparency in the study design, description of acceptability and feasibility framework, recruiting strategy is clear and inclusion and exclusion criteria well described. Data analysis is strong, with detail on the approach, how coding was handled, how themes developed, and discussion of data saturation, improving the credibility of the findings. However, most healthcare providers interviewed were from private clinics, and patients interviewed were recruited through social media sites, suggesting they were already active online. This may introduce bias into the data and reduces transferability and validity.
Hohmann-Marriott 2023 [24]	New Zealand	Understand the role that menstrual apps can perform in healthcare	Thematic analysis of online qualitative survey and focus groups with expert stakeholders *P* = 144 survey responses *P* = 10 focus group participants	Participants felt fertility apps were helpful for managing reproductive conditions and improving communication between providers and patients, but reported concerns about inaccuracies and app limitations.	X	Strong transparency on overall approach and how feedback was incorporated for who didn’t have time for a focus group but wanted to contribute. However, only half of survey respondents had actually used a fertility tracking app reducing application to our research question, and the small number of participants limits generalisability. Purposive sampling was used, but the researchers generated a list which may introduce bias and was not discussed in the limitations. Sata saturation is not discussed and only half of the data is discussed, limiting reliability and completeness of emergent themes.
Homewood 2020 [25]	Sweden	Gather feedback on user design of Ovum fertility tracker through a shared, domestic experience	Interactive Design Study *P* = 18	The saliva-based fertility-tracking tool created uncertainties among participants who reported confusion and inaccuracies during use.	X	Strong methodological rigor, with discussion of relexify around the emotional involvement of the research team, increasing credibility. Transparent description of thematic analysis, strong presentation of data, and contradictory data discussed. However, the authors created the device tested in the study, increasing risk of researcher bias. Recruitment is also not transparent; the author chose 9 out of 22 couples but does not explain why, increasing risk of researcher bias. Very small sample of participants and a high drop-out rate, limiting the transferability and generalisability of the study.
Jones 2015 [26]	United Kingdom	Get a deeper understanding of the experiences of women using home digital ovulation tests wishing to conceive	Framework analysis of telephone interviews with women *P* = 36	Women reported the use of digital ovulation trackers at home helped to improve understanding of their fertility cycle and when to try to conceive, but could also be burdensome and difficult to understand.	✔	Methodological rigor underpinned by strong justification of qualitative methods, use of randomised groups, and description of why participants were interviewed. Strong description of analysis process, description of coding framework, rich data to support findings and themes, discussion of conflicting data and how data saturation was reached increasing the transparency of data analysis. Recruitment was through the Clearblue UK website, suggesting those recruited were already familiar with home ovulation tests, which introduces selection bias and reduces transferability.
Lerma 2018 [27]	India	Assess user satisfaction and acceptability towards a cell phone-based SMS application for fertility awareness	Thematic analysis of focus groups with women seeking family planning services *P* = 21	Participants found text-based fertility tracking to be helpful, private, secure, and convenient.	X	Study design is mainly quantitative, with limited qualitative data, reducing application to this review. There was little transparency or methodological rigot in data collection, lacking details on the focus groups, how data was recorded, how consensus was reached, and how data was analysed. The study did not specify which participants utilised the tool to prevent pregnancy, rather than trying to conceive, making it difficult to apply results to this review and limiting transferability.
Mathiason 2023 [28]	United States	A review of Femtech technologies (LOONCUP, Glow, Clue) to suggest feminist engineering improvements	Case study analysis *P* = 2 tools	All three case studies of existing Femtech technologies present significant shortcomings in reinforcing gendered responsibilities and norms, data privacy, and user-built goals.	X	Provides strong justification for a qualitative study design, theoretical approach, and case study selection. However, a single author completed the analysis, increasing chance of researcher bias and reducing the credibility of the findings. Additionally, the description of ontology and methodology are incomplete, analytical framework unclear, and many citations are from newspaper articles or popular media, further reducing credibility. The inconsistency in how each case study is discussed, risks introducing researcher bias.
Mu & Fehring, 2023 [29]	United States	Pilot evaluating and comparing the length of the fertile window using a fertility tracking app with the Clearblue Fertility Monitor (CBFM)	Mixed-methods pilot study evaluating and comparing whether Clearblue ovulations tests, in combination with Premom or Easy@Home apps, help users understand their fertility *P* = 30	Participants reported mixed feelings towards at-home ovulation tracking apps. They found it overall easy to use, but sometimes found interpretation challenging.	X	The study only assessed current users of ovulation-tracking which opens the results to bias and decreases transferability. The qualitative reporting in the paper was limited, and only a few quotes from participants were included, reducing transparency and the credibility of the themes. Little transparency on the methods of data collection, analysis, how coding was conducted, and whether saturation was reached, reducing the rigor.
Novotny & Hutchinson 2019 [30]	United States	Feminist analysis of technical communication in Glow	Feminist critical analysis of application language, Terms of Service, and Privacy Policy *P* = 2	Glow provides a unique service to users who otherwise may not access fertility care services, but also poses potential risks and pressures on vulnerable populations.	X	The study has a strong justification of methodological approach, theoretical framework is well described, and approach is based in existing scholarship, demonstrating rigor. Though the analytic framework well described, but coding and thematic analysis unclear, reducing the transparency of data analysis. Theoretical framework is only applied to one app (Glow), limiting generalizability and transferability of findings.
Parry et al. 2022 [31]	United States	Debate whether telehealth is a valuable resource in reproductive endocrinology and infertility	Opinion piece from reproductive endocrinology and infertility providers *P* = 6	Telehealth will continue to play an integral part of reproductive and infertility care, but providers must stay vigilant to ensure it does not widen existing disparities or place undo burden and risk on patients.	X	The article recruited representatives with a range of opinions to improve the neutrality of presented views. However, its opinion-based; all authors are practicing reproductive health professionals which may bias their opinions. No methodology or data analysis is described since this is an opinion piece published in a peer-review journal, included as grey literature to capture diverse professional viewpoints.
Patel 2024 [32]	United Kingdom	Identify the experiences of users of period tracking apps in relation to which apps they use, their frequency of use, the types of data, and their attitude to the apps	Observational mixed-methods study of people who have used a period-tracking app *P* = 375	Participants reported a wide variety of needs and use of menstrual-tracking apps. Those using the apps to conceive reported concerns about inaccuracies and effectiveness of tools.	X	Methodological rigor is demonstrated with a well justified and described thematic analysis, data saturation being reached, and a transparent explanation of how qualitative data was collected. However, recruitment was via the researcher’s social media accounts, introducing the risk of sampling bias. Coupled with only 61.9% of participants using a menstrual-tracking app, it also restricts the generalisability of findings and brings into question the appropriateness of the original study’s design, limiting its relevance to the review.
Reime 2023 [33]	Denmark	Analyse the normativities of reproductive bodies through assessing Clue, Tilly, and Drip	Critical analysis of app language using the walkthrough method *P* = 3	The three platforms analysed perpetuate existing heteronormative pressures and expectations.	X	Strong description of the methodological approach and ‘think aloud’ approach, and data collection methods are transparent. The authors exhibit strong reflexivity and discuss how their lived experiences impacted their findings. However, they also acknowledge significant limitations to the transferability of findings given their positionalities. There is a risk of researcher bias given the authors are offering their subjective walk-through feedback on the applications. Although the authors reach consensus among themselves, there is limited data saturation which reduces the credibility of the findings and completeness of emergent themes.
Yousef 2021 [34]	Jordan	Explore the perceptions of women in Jordan use of digital health technology to support access to fertility planning services	Thematic analysis of interviews with women seeking family planning services *P* = 32	Participants reported at-home digital technologies would be feasible, cost-effective, well-accepted, and beneficial in increasing women’s understanding of their reproductive health, so long as they were appropriately combined with clinical care.	X	The studies shows strong methodological rigor by providing strong justification of qualitative approach, details about the topic guides, probes, settings, coding consensus, and saturation of data. Triangulation is reached and strengths and limitations of the study clearly noted, increasing validity and credibility. Patient participants were already attending in-person health centres, so findings may not apply to those without access to medical services, reducing transferability. The study did not specify which feedback which applied trying to conceive versus preventing pregnancy, making it difficult to apply some of the results to this review.

## Results

**Fig. 3:**
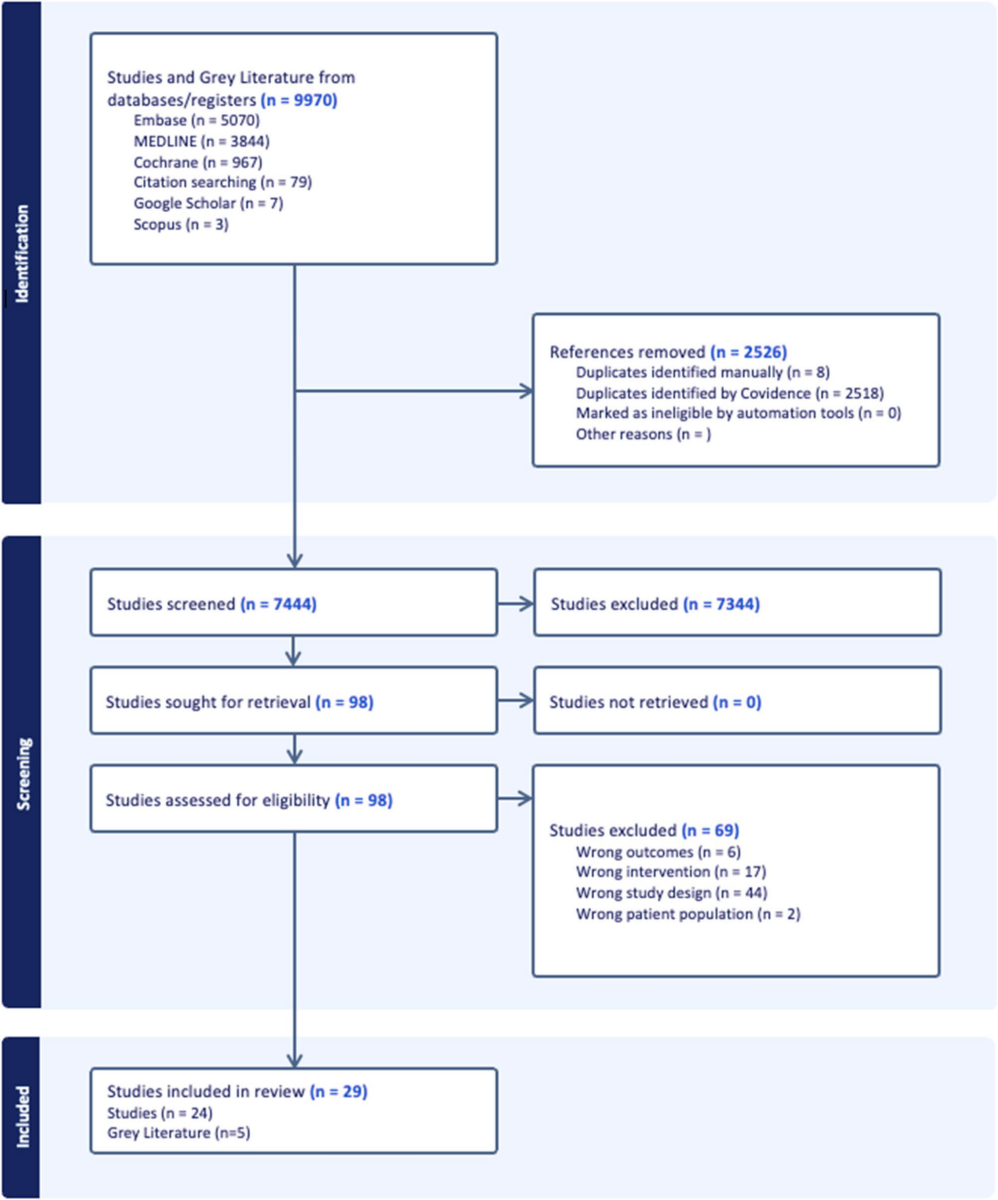
PRISMA flowchart [35]

The database search identified 9,970 studies for screening. 2,518 duplicates were identified and automatically removed by Covidence, and 8 duplicates were identified and removed manually. This left 7,444 study titles and abstracts to be screened. Of these, 97 full-text studies were further screened for eligibility, and 69 studies were excluded (see Fig. 3, above). One study was rejected because the only resulting publication available was a conference poster which did not contain qualitative material to analyse, and no further report or publication was provided by the author. Most papers were published in the United States and United Kingdom, and the remainder published across mainly high-income countries, specifically Canada, Denmark, New Zealand, Sweden, India, and Jordan. They included a diverse mix of qualitative and mixed methodologies, aims, and both journal articles and grey literature (see Table 1 for more details). Though most of our search terms were not gender-exclusive, the literature available mostly referred to cisgender, heterosexual women. A thematic analysis generated eighty descriptive codes from the content which were iteratively refined into sub-themes and then two major themes with discussion between authors; “positioning digital trackers in fertility healthcare”, and “conflicts and disruption” (see Table 2 for a theme overview).

**Table 2:** Descriptive themes across the literature

Study Author	Descriptive Themes								
	Wide awareness/ use of fertility tracking apps	Improved Communication/ Advocacy with Providers	Natural Approach	Trustworthiness/ Inaccuracy	Mixed sense of benefits/risks	Burdensome	Reinforces gender roles	Privacy concerns	Standardization
Allard-Phillips et al. 2023 [8]	✔								
Bench-Capon et al. 2018 [9]		✔		✔	✔				
Blair et al. 2021 [10]	✔				✔				
Costa Figueiredo et al. 2017 [11]	✔	✔	✔		✔	✔			
Costa Figueiredo 2018 [12]					✔	✔			✔
Costa Figueiredo 2020 [13]				✔	✔	✔	✔		
Costa Figueiredo 2021 [14–16]	✔	✔		✔				✔	
Costa Figueiredo 2021 [14–16]		✔			✔	✔			
Costa Figueiredo 2021 [14–16]	✔	✔			✔		✔		✔
Costa Figueiredo 2024 [17]	✔	✔		✔	✔				
Donelle 2021 [18]		✔		✔					
Freilich 2022 [8]					✔		✔		
French 2022 [19]		✔		✔		✔			✔
Gambier-Ross 2018 [20]		✔		✔	✔			✔	
Grenfell 2021 [21]		✔	✔	✔	✔		✔		
Hamper 2020 [22]			✔	✔	✔		✔		✔
Hamper 2022 [14]	✔		✔			✔	✔		
Harrison 2023 [23]		✔		✔	✔				
Hohmann-Marriott 2023 [24]		✔		✔	✔				✔
Homewood 2020 [25]				✔	✔				
Jones 2015 [26]			✔		✔	✔			
Lerma 2018 [27]									✔
Mathiason 2023 [28]				✔		✔	✔	✔	
Mu & Fehring, 2023 [29]					✔	✔			
Novotny & Hutchinson 2019 [30]	✔	✔			✔	✔	✔	✔	
Parry et al. 2022 [31]		✔			✔			✔	
Patel 2024 [32]		✔	✔	✔	✔			✔	
Reime 2023 [33]					✔		✔		✔
Yousef 2021 [34]		✔		✔	✔	✔		✔	

### Positioning digital trackers in fertility care

The first major theme concerned the position of trackers prior to, in combination with, and in place of clinical fertility treatment provision. This theme grappled with whether new digital technologies fall within or outside of traditional clinical care; in some cases, they are described as alternatives to more invasive or medicalised fertility care. In others, they are positioned as a new method of accessing, advocating, and communicating with fertility specialists.

#### Prior to seeking medical care

In some cases, patients adopted digital tools as a cheaper and easier step towards trying to conceive without having to resort to more invasive options [9–11, 14, 36]. For example, Costa Figueiredo’s interviews with women facing challenges conceiving in the USA note a participant who “didn’t get pregnant for quite a few years.” So, she started “trying to find the reasons that I can probably change […] to help the process.” [14]. Digital trackers were often framed as “non-medical” ways of increasing the chance of conception, and if the trackers proved ineffective, users then considered escalating their case to clinical care [11]. The sense of responsibility for one’s own health and fertility is reflective of many women’s journeys into digital fertility tracking when they begin planning for a pregnancy, or find themselves struggling to conceive. Women reported using apps or web trackers to “see if there’s anything else that I could or couldn’t be doing that might sort of increase chances” or to try and identify whether there is something “wrong” with their bodies that would require medical escalation [10, 15, 26].

#### Digital tracking alongside clinical services

Several studies described women using trackers alongside fertility care to communicate or advocate to healthcare providers or for “regaining control from doctors when [they] felt they were not being heard” [11, 12, 26]. Some patients understood this as a collaborative measure to help their doctor diagnose them: “‘I did [tracking] for my doctor’s sake so I used the app’” [19, 20]. This was especially true for conditions that were seen as tricky to diagnose such as endometriosis, polycystic ovary syndrome (PCOS), or unexplained fertility, where trackers were used to record symptom data to assist with diagnoses [15, 24–26, 32]. In other cases, women felt presenting their doctors with tracking data was necessary to receive adequate care: “At my age [41] I don’t necessarily believe that doctors will take the specifics of my case seriously.The apps.help me to demonstrate I’m still worth taking seriously as a fertility case” [16] Patients also described using tracking data to “convince” doctors that their claims were credible and they should be escalated to fertility services, either by using longitudinal symptom tracking, cycle tracking, or to demonstrate how long they had been trying and timing sex [21, 24].

The literature suggests that women continue to utilise fertility tracking platforms even once they meet with providers or start fertility treatments, and that some providers recommended their patients begin using tracking apps to assist with their treatment [8, 11, 14, 21, 33]. Figueiredo’s interviews with health providers in the United States included professionals who were eager for their patients to adopt these technologies: “It’s good to have well-informed patients who understand their body…I think that these apps can help educate patients very well and they can be very helpful” [14]. This also applied to providers in low resource settings; Youef et. al’s interviews with midwives in Jordan and Lerma et. al’s interview with women in India reported providers and patients found tracking technologies to be empowering and saw them as effective [27, 34].

Providers and researchers also reported that digital tools could provide patients with support, expectation management, and a better understanding of fertility data [18, 20, 23, 24, 30, 31]. Receiving guidance or education digitally allowed patients to review materials in their own time and gave them space to weigh difficult decisions: “It would be so helpful as I can take my time reading” [34]. However, the need for consistent educational messaging was brought up across several studies, with one participant in Harrison’s interview noting “…as long as there’s consistency (between [app from study] and clinic information), that’s what we need above and beyond anything else is consistency of information” [23]. In some cases, tracking also improved or expedited treatment by providing a more accurate and longitudinal history of a patient’s menstrual cycle [14, 24, 30], increasing patient-provider communication [24, 31], providing a more robust understanding of symptoms and hormone levels [14, 24, 31], and helping organise and coordinate fertility services by tracking appointments [31]. Health providers, authors, and patients across the literature reported that it was important for clinicians to be aware of and open to digital fertility trackers, and to discuss the use of these applications with their patients [8, 9, 12, 18, 20, 24, 29, 30, 36].

#### Digital tracking in place of clinical services

Though the most common sub-theme in the literature was the use of fertility trackers alongside clinical care, the third sub-theme identified how trackers could be utilised to avoid the clinical system. Many tools were adopted because this system was difficult to access. For example, French et al.’s interviews with women using the Natural Cycles fertility tracker reported participants in the UK were anxious about overburdening the NHS, and that health professionals would not be “interested” until they had been trying to conceive for over a year, or were more than 12 weeks pregnant [9]. Grenfell’s article on these interviews also reported women with fertility concerns felt dismissed by the health service, or “unjustified” given NHS resource constraints [21]. Cost and lack of access also factored into the use of trackers, both in US and UK-based studies [11, 16, 21, 28, 30]. For example, Novotny and Hutchinson’s critical analysis argued that the tracking app Glow provided unique and affordable services to healthcare demographics that could not otherwise pay for professional fertility services [30].

In some cases, participant’s “pull towards natural” [37]was due to personal preferences around privacy or partner preferences [11, 12, 26, 36]. Grenfell’s interviews with women using the Natural Cycles app found women preferred these due to negative experiences with hormones, the physical and mental burden of IVF, and the feeling that technology was more “natural” compared to biomedical intervention: “I just didn’t like the idea of pumping myself full of.chemicals.I wanted to use something as natural as possible. I thought this seemed a bit more scientific” [21].

The literature suggests male partners do not always see trackers as ‘natural’, but also may expect women to conceive without medical intervention, leading women to use the tools secretly or choosing less-clinical apps [12, 26, 36]. In other cases the apps were something individuals kept to themselves, or couples only shared with each other: “my husband will make sure that no one except me and him know about using the mobile application”; “I didn’t really want to ask anybody” [10, 34].

Some participants leant away from specialised medical interventions due to inconclusive or negative experiences within the fertility industry [14]. Participants in Jones’ interviews with women using home ovulation tests partly credited this to lower levels of distress in tracking compared to fertility procedures [26]. For others, distrust, lack of communication, or a failure to get a diagnosis or treatment had driven them from health services to digital solutions. As one participant in French’s interviews stated: “I trust my body at this point more than I trust the advice or input that I’m getting from my doctors… this is something that I’m doing for myself because I’m not being listened to.” [9].

Across the literature, women reported similar pivots after receiving incomplete medical advice, lack of medical support following traumatic experiences like miscarriages, broad “unexplained infertility” diagnoses without further options for treatment, or dismissal from doctors [11, 12, 14, 18, 21, 28, 37].

### Conflicts & disruption

The second major theme concerned how the design, use, and implementation of digital fertility trackers created conflicts between patients, providers, and disruption across the greater fertility industry.

#### Conflicts over the role and limitations of trackers

Many tracking tools in the included studies relied on standardized experiences of menstruating, ovulation, and sub/infertility to make predictions and provide guidance to users. The same technology beneficial for identifying irregularities that could lead to diagnosis or help escalate a patient to clinical care also created anxiety for users around whether their data was “normal” (‘my body is not consistent with the data’ [12]), and frustration for users whose normal experiences with irregular cycles due to PCOS, endometriosis, or unexplained infertility were excluded from the algorithm (‘More focused on normal bodies not really with health related i.e., pcos’ [15, 22, 24, 33, 36]. Standardized algorithms often failed to consider the impact of infertility or fetal loss on menstrual cycles, reminding users they were still not pregnant or had lost a pregnancy [22, 24].

Sometimes conflict stemmed from disagreements between patients and providers on how tracking technologies should be used and what data was valuable, especially when app guidance directly conflicted with clinical guidance. For example, basal body temperature (BBT) is a common data point used by apps as a method of ‘predicting’ ovulation by measuring slight temperature changes caused by increases in progesterone during ovulation [17]. However, BBT charting is often ineffective because body temperature is influenced by many factors, including some which coincide with infertility, such as stress and hormone imbalances. Participants across multiple studies had been told by clinicians BBT was an unreliable and unhelpful measurement:

“I’ve been to a gynaecologist and they’ve said that basal temperature is not the best way to record fertility and they’ve moved away from that a long time ago, which was slightly disconcerting… I said I was a bit surprised because I’d bought this app and it’s medically certified and they use basal temperature, that’s what I’ve been doing” [21].

Yet participants still used or considered using it for tracking purposes: “[my doctor] said it really wouldn’t be worth the effort…I have been thinking about doing it my next cycle just to see what happens though” [11, 14, 26, 38]. Women also disagreed with provider guidance on when to have intercourse, the practicalities of UK NHS guidance to have sex every 2–3 days, the reliability of ovulation prediction kits (OPKs), and what medications should be taken to encourage ovulation, all of which were influenced or connected to their use of digital technologies [11, 14, 26]. Participants also repeatedly mentioned ovulation tracking predictions were incorrect or conflicting with provider estimates when compared to ovulation test results in multiple studies: “I never ovulated, according to the software… but I still got pregnant.” [11, 14, 25, 36].

#### Decentring the clinic

Several studies reported fears of unintended consequences on workflows [23, 31], erosion of care [31], privacy and security [28, 31, 32, 34], responsibilities of “health care work” getting delegated to patients [18, 21, 23], and unreliable or inaccurate fertility predictions or measurements [21, 25, 32, 34]. Many authors discussed how the burden of interpreting or acting on tracking data without assistance from professionals risked putting patients in positions of danger by creating a false sense of confidence [13, 18], gendered labour [18, 21, 25, 28], self-blame and stress [23, 31], requiring data interpretation without sufficient guidance [18, 21, 23, 34], and unwitting access of low-quality online care [31, 34]. Providers also feared digital apps could increase appointment times, give patients unrealistic expectations of treatment success, or create challenges in practice by supplying patients with untrustworthy or out of date information [16, 23, 24, 30, 34]. This is highlighted by a provider in Hohmann-Marriott’s focus group interviews in New Zealand: “you’re only gonna get out of your app, what you put into it…if you’re not inputting the right information or enough information, it can’t tell you anything” [24].

#### Disrupting care delivery

Contrarily, several articles also discussed how these tools could disrupt the industry in ways that benefit or reimagine physician workloads and care pathways [23, 28, 30, 31]. Home testing solutions in particular were appreciated by health care professionals for providing effective, real-time fertility monitoring at home with minimal obstruction to a participants schedule or physiological state [22, 24]. It was also noted that virtual visits and remote monitoring could save both patients and health care professionals time, money, and childcare costs compared to commuting to in-person appointments, something a physician-written opinion piece stated is already “the reality for several patients at many centers in the United States” [31, 34]. US-based articles also discussed the economic value of digital tracking in the for-profit fertility industry, including redeemable payouts to local fertility clinics for app subscribers, partnerships between fertility apps and private fertility clinics, and apps that claimed to offer more affordable fertility tests or cheap ad-supported video consults for un- and under-insured patients [28, 30, 31].

In comparison, UK studies interviewing patients and providers on paid apps or apps that were partnered with private fertility clinics found participants more cautious about the costs of these services [9, 18]. In French’s interviews with patients in the UK using the “Natural Cycles” app in an effort to conceive, some participants felt the app was more trustworthy than private pharmaceutical companies “selling you a drug”, and felt the app should perhaps be provided freely through the NHS to decrease barriers to access [9]. Additionally, Harrison et al.’s feasibility study of using an assisted reproduction technologies “planning” app at the beginning of treatment found both providers and patients speculated patients may be cautious about any approaches or tools could appear to be profit-driven: “They [patients] might just feel that… it’s under the NHS and my free round and the clinic probably wants me to spend more money’” [23].

## Discussion

The literature suggests digital tools are used throughout care pathways; prior to approaching medical services, during treatment, and sometimes after unsatisfactory treatments end (though not necessarily linearly). Both patients and providers felt digital trackers could empower individuals trying to conceive, improve delivery of care, and be used by patients to advocate in clinics. However, while the use of these technologies was convenient for some individuals and clinicians, there are still significant challenges they introduce to both patient-provider relationships and the broader fertility industry. The shift of responsibility for tracking and decision-making these tools towards patients these tools create has resulted in patients receiving conflicting health information that can cause confusion and instigate distrust in medical systems and guidance.

This review has several limitations that may reduce the strength of our findings. First, all included articles were written in English, which may have excluded relevant material in other languages. This also may have impacted the range of countries included in the review, given most of the studies were published in Western countries. Additionally, as this is an emerging field, there were limited studies that met our inclusion criteria, we did not exclude any articles based on quality, and a high percentage of articles and grey literature were published by either Costa Figueiredo or as part of the Freja project. Several of the studies included were also funded by fertility tech companies, which may introduce commercial bias into the results. We were cautious to ensure the themes and sub-themes described in our review were present across a range of literature and authors to reduce the risk that data from these two sources were not over-represented or resulted in homogenous findings. As mentioned earlier, although we strived to make most of our search terms gender-inclusive, the literature included largely focuses on cisgender, heterosexual women which limits the generalisability of our findings. The included literature also varied methodologically and disciplinarily, which created challenges when applying our quality framework and interpreting results.

Our review has implications for both medical practice and future qualitative research; provider awareness was a key expectation from both patients and providers in the literature, which will require appropriate training and more engagement between platform developers and the medical profession. While some studies explicitly investigated areas where digital trackers could assimilate into existing care pathways (i.e. similarities and differences between patient and provider data needs), more work is needed to understand how this can be done without burdening clinicians or complicating patient-provider communication [14]. Additionally, the pattern of patients turning to digital platforms in lieu of satisfying medical treatment needs to be better understood, both in an effort to improve patient experiences and understand consequences of seeking medical direction and advice from (potentially) less reliable sources.

Though recent research suggests the use of menstrual trackers to try and conceive is rapidly expanding globally, there is limited information available on the consequences for medical practice [39]. The evidence of fertility trackers being used in combination with healthcare services synthesised for this review was often only a small theme or partial focus of the collected studies, rather than the central research question. The data on the application of fertility trackers for clinical care is also distorted by both low quality and evidence-based, clinical tools getting falsely compared. Guidance and research that differentiates trackers that are developed based on a strong evidence-base from other tools would be valuable in clarifying what types of digital interventions are useful and applicable for clinical care. App development companies have also called for this distinction, voicing frustration with academic publications that lump all “fertility awareness-based” apps together [40]. Additionally, future research explicitly examining fertility trackers in the clinical context would be helpful in creating a more comprehensive understanding of their use and applicability. The narrow range of authors, countries of origin, lack of inclusivity, and available studies reflected in this review generally suggests there is much more work to be done in this space. Given that about a fourth of the included literature was funded by commercial entities, additional research investment from impartial sources would be imperative to balancing the evidence around these tools.

## Conclusion

This synthesis demonstrates that digital fertility trackers are being used by people throughout their clinical fertility journeys as a convenient way to seek information, advocate and communicate to providers, and organise information about their reproductive health. While the majority of the literature reviewed suggests people use these tools alongside medical assistance from health professionals, some individuals are also turning to digital tracking technologies in place of clinical care. The range of technology available, how platforms are used, whether use is tacit or hidden, engagement of providers, and degree of integration with clinical care differs significantly across the literature. More research is needed to understand how digital fertility tracking applications can be better integrated into clinical care pathways to improve the experiences of both users and providers, particularly with respect to patient-provider relationships and trust. Additionally, the inconsistency of device quality, privacy, and usefulness highlights the need for improved guidance from regulators, both to ensure patient safeguarding and increase their potential impact on fertility care outcomes.

## Data Availability

No datasets were generated or analysed during the current study.
